# Social-Ecological measure of resilience: an adapted measure for Persian-speaking university students

**DOI:** 10.34172/hpp.2020.34

**Published:** 2020-07-12

**Authors:** Mohammadali Amini-Tehrani, Mohammad Nasiri, Raheleh Sadeghi, Elahe-Sadat Hoseini, Tina Jalali, Hadi Zamanian

**Affiliations:** ^1^Department of Psychology, Faculty of Psychology and Education, University of Tehran, Tehran, Iran; ^2^Health Psychology and Behavior Medicine Research Group, Student Scientific Research Center, Tehran University of Medical Sciences, Tehran, Iran; ^3^School of Health, Qom University of Medical Sciences, Qom, Iran

**Keywords:** Self-assessment, Ecology, Psychometrics, Psychological resilience, Young adults

## Abstract

**Background:** There is no validated instrument for Persian-speaking students to apply the social-ecological resilience theory (SERT), which emphasizes the ecological resources for developing resilience. The study aimed at developing the student social-ecological resilience measure(Student-SERM) in Iran’s context.

**Methods:** Three separate samples of undergraduates participated in this mixed-methods research from the University of Tehran, Iran. Phase-1 qualitatively explored the resilience features in the university setting, to devise the university-specific subscale (USS). Phase-2piloted the construct validity and reliability of the Student-SERM in 242 undergraduates, who also completed Depression, Anxiety, and Stress Scale (DASS-21). Phase-3, as a cross-validation study, investigated 487 undergraduates, who completed the refined Student-SERM, HospitalAnxiety and Depression Scale (HADS), and three indices screening academic performance, loneliness, and suicide acceptability. Exploratory factor analysis (EFA) and confirmatory factor analysis (CFA), Pearson’s correlation, and Cronbach’s alpha were performed.

**Results:** Phase-1 yielded nine items for USS. In phase-2, EFA indicated the construct validity of the main 20-item measure (RMSEA=0.06 and SRMR=0.04) and the nine-item USS (RMSEA=0.07and SRMR =0.04), and the reliability and convergent/divergent validity were confirmed. In phase-3, EFA (RMSEA=0.07 and SRMR=0.04) and CFA (RMSEA=0.07, CFI=0.89, TLI=0.87,and SRMR=0.07) in two separate subsamples and CFA (RMSEA=0.06, CFI=0.92, TLI=0.90,and SRMR=0.06) in the total sample indicated the construct validity of the refined Student-SERM, including family, peer, culture, growth, and USS subscales. The reliability and convergent/divergent validity were also reconfirmed.

**Conclusion:** The Student-SERM incorporates ecological resources, accounting for the students’resilience. Since the resilience process involves a return to healthy functioning after adversity, further research can examine the application of Student-SERM in high-risk student populations.

## Introduction

Resilience is the process of recovering from highly stressful situations or traumatic experiences, which increase the risk of developing psychological and adjustment disorders.^1^ According to the Kurt Lewin’s field theory, Ungar^2^ has proposed the social-ecological resilience theory (SERT), which considers resilience as a function of an individual’s interaction with the environment. According to the Ungar’s theory, resilience is a set of behaviors, related to adaptive outcomes (e.g., graduation from high school, relationship with prosocial peers, and developing self-esteem) in an adverse context.^2^ These behaviors are partly determined by personal strengths and challenges, which depend on the individual’s ecological context. In addition, resilience is characterized by two other elements: 1) opportunities for the individual’s development, which may vary in terms of availability and accessibility in a given ecological context; and 2) the meaning of resilience, which is based on the individual’s cultural context and varies relative to the social structure.^2,3^

To operationalize this new concept, the 28-item Child and Youth Resilience Measure (CYRM) was developed for individuals aged 13-23 years.^4-6^ More than 1400 young people, living in high-risk environments in 11 countries, participated in a pilot study to design this instrument. The personal, peer, family, and community resources constituted the item pool.^5^ Also, Liebenberg and Moore^7^ developed the 28-item Adult Resilience Measure (ARM),^8^ based on CYRM for individuals above 23 years. The ARM includes five socio-contextual components, that is, social/community inclusion, family attachment and support, spirituality, national and cultural identity, and personal skills/competencies.^7^

There is merit in using SERT for different populations, including university students, who are at the age of transition to adulthood. Firstly, the focus of employment, science production, mental health, and public policies is on students, as social individuals with different personal, familial, and communal aspects.^9,10^ Secondly, students are regarded as valuable resources in both developing and developed countries, and student life is associated with personal, familial, and community growth.^11,12^ However, multiple studies, addressing the mental health and behavioral concerns of students, found that depression, anxiety, suicidal behaviors, and academic performance were the most important themes.^13-15^ Although university students may have a better mental health than their same-aged non-collegiate peers,^16^ their pre- and post-matriculation psychological problems may increase the risk of poor academic performance and university attrition.^16,17^ Therefore, it seems necessary to approach the students’ healthy functioning in a way that their contextual resources and personal capabilities are considered.

The fact that student life marks the transition from adolescence to adulthood indicates its growth-oriented nature.^18^ A student is an individual, who primarily attends university or college to find personal, educational, and skill development opportunities. Also, “student” is an umbrella term, encompassing individuals with different strengths and challenges, who share a particular environment (i.e., academic settings). Overall, the student’s life explicitly reflects the socio-ecological context. Therefore, the SERT framework is deemed competent to define student resilience.

On the other hand, resilience research using available instruments has consistently limited the resilience of students to their characterology and personal strengths.^19-21^ Contrary to the mainstream position, Ungar,^2^ in a review study, found that personal traits cannot explain the effects of contextual factors, which are known to play a critical role in recovery from stressful events. According to SERT,^2^ the influence of nurturing factors is greater than that of inherent factors. Therefore, environment and environmental resources play a more significant role in the resilience process, compared to personality, cognitive performance, and personal resources.

The present study aimed at designing, validating, and examining the psychometric properties of the student social-ecological resilience measure (Student-SERM), adapted from CYRM^5^ and ARM,^7^ according to a SERT framework. This study was conducted in Iran, where collectivism and cultural factors highlight the growth and excellence of youth at the forefront of government planning and family concerns. Thus, the post-positivist perspective^2^ underlying the instrument could be seen well-suited for Iran’s context, because it holds the different layers of influence independent, yields the content with a global nature, and embraces the specific features of Iran’s developing context.

## Materials and Methods

### Study design and sample

This mixed-methods research included one qualitative and two quantitative phases. Three groups were independently recruited from University of Tehran, Iran. As suggested in the manuals of CYRM^6^ and ARM,^8^ SERT provides an opportunity to develop a site-specific subscale for assessing the main features of resilience in a target population in a specific setting (i.e., university). Accordingly, in phase 1, the qualities of university students, who may manifest the key features of resilience in an academic setting, were qualitatively explored. For this purpose, eight students (one female PhD candidate, two male graduate students, one female graduate student, three male undergraduates, and one female undergraduate) were interviewed. The interviews explored the features of academic settings, which might enhance the students’ physical, mental, social, and spiritual health. The respondents were also asked about their definition of a resilient student, based on their own personal or vicarious experiences.^6,8^ The interviews started with an open discussion about the socio-ecological significance of resilience to familiarize the subjects with the topic. Subsequently, a new subscale was incorporated in phase 2 for quantitative analysis.

Phase 2 was a pilot study with a cross-sectional design, conducted from December 2017 to February 2018. This phase aimed at examining the construct validity (factor structure), reliability, and concurrent validity of the Student-SERM quantitatively. The study included 242 undergraduate (n = 171, 70.7%) and graduate (n = 64, 26.4%) students from different faculties. The study population consisted of 114 (47.1%) males and 125 (51.7%) females, aged 19-29 years. On average, the students had completed six semesters at the university.

Finally, phase 3 was carried out with a cross-sectional design from April to May 2018 to cross-validate the refined version of the Student-SERM, according to phase 2. The sample included undergraduates from three fields of “humanities” (five schools), “engineering” (mixed schools), and “basic sciences” (two schools). A total of 524 undergraduates received the questionnaire booklets in the classrooms; they were instructed to return the booklets in sealed envelopes after completion. However, 37 incomplete questionnaires were excluded; therefore, the final sample size was 487 (response rate = 92.9%).

### Data collection

In phase 1, some semi-structured, open-ended questions were asked from the students, according to the CYRM and ARM manuals^8^ to conduct the interviews.^6^ The first author conducted the interviews, which were then audio-taped and transcribed. In phases 2 and 3, data were collected based on a self-report method, using the Student-SERM and measures of convergent/divergent validity.

### Resilience measures

*CYRM*

This tool, as the primary measure of social-ecological resilience, was introduced in the International Resilience Project (IRP) by the Resilience Research Center (RRC) in a mixed-methods research on 13- to 23-year-old individuals across 14 countries.^4^ It addresses the individual, relational, cultural, and communal resources of resilience and encompasses three factors of “individual capacities” (i.e., personal skills, social skills, and peer support), “primary relationships” (i.e., caregiver’s physical and psychological caregiving), and “contextual factors” (i.e., spiritual, cultural, and education).^5,6^

*ARM*

This scale was developed based on CYRM for adults aged above 23 years.^7^ The 28 items of CYRM have been validated in adult survivors of childhood maltreatment.^7^ This scale contains five major subscales of “social/community inclusion”, “family attachment and support”, “spirituality”, “national and cultural identity”, and “personal skills/competencies”.^7,8^

### Translation and adaptation

The Student-SERM contains the items of both CYRM and ARM. The age range of the target population was 18-29 years in this study, and their general features were as follows: 1) developing personal capacities; 2) primary reliance on family for support, while trying to become independent; 3) living a single life, while having an intimate partner; and 4) considering university education as a socially prioritized responsibility in life. Three categories of items were included in the Student-SERM to address the ecological context of the target population:

(I) The original Student-SERM consisted of 30 items, including 26 items from CYRM and four items from ARM (“having people to respect”, “sense of belonging to the community”, “importance of skill development”, and “having an opportunity to apply the acquired skills in real life”). (II) Three separate items were also included in the Student-SERM to independently address “partner support”, as we decided to distinguish the individual’s family life from his/her social/intimate relationships. Based on the CYRM and ARM, the items focused on “talking about feelings”, “receiving support during difficulties”, and “enjoying the partner’s culture and tradition”. (III) Nine items developed in phase 1 were included as a separate subscale. All items were rated on a five-point Likert scale: “not at all” (1), “a little” (2), “somewhat” (3), “quite a bit” (4), and “a lot” (5). There was no reverse-scored item, and the total resilience score was the sum of the item scores.

The designed instrument was translated using the forward-backward method. The first and fifth authors, who are qualified English-to-Persian translators and editors, translated the scale into Persian. The translated draft was prepared based on the two initial translations. It was then back-translated into English to ensure semantic and conceptual equivalence; finally, the Persian version of the Student-SERM was approved. It was then presented to ten students, including two PhD candidates, four graduates, and four undergraduates to examine the eligibility and intelligibility of the items. No issue was raised about the wording or legibility of the final translated version.

### Convergent and divergent validity 

The Depression, Anxiety, and Stress Scale (DASS-21) was employed to assess the divergent validity of resilience variables in phase 2 of the study.^22^ Each subscale entails seven statements, assessing the individual’s subjective experience in the past seven days. This scale has been validated in the Iranian population.^23^ Also, the Hospital Anxiety and Depression Scale (HADS)^24^ was used to assess the divergent validity of resilience variables in phase 3. This scale consists of 14 items, scored based on a four-point Likert scale. Each subscale entails seven statements, examining the individual’s symptoms in the past seven days. This scale has been validated in the Iranian population.^25^

In phase 3, three indices were used to screen the psycho-socio-educational status of the participants. The“feeling of loneliness” (FoL)was assessed by a single-item index to determine the degree to which the respondent felt lonely in the past year. This item (“I have felt lonely over the past year”) was scored on a five-point Likert scale (“never”, “rarely”, “often”, “more than often”, and “always”), with higher scores indicating a higher FoL. The “perceived academic performance” (PAP) was also evaluated using a single-item index (“How do you evaluate your educational status?”). This index was rated on a five-point Likert scale, ranging from “very bad” to “very good”, with higher scores indicating a better PAP. Moreover, “suicide acceptability” (SA) was assessed by a single-item index (“It is argued that suicide is a good choice to become free from pains and sufferings. Based on your opinion, suicide is…”. This item was rated on a five-point Likert scale (“completely unacceptable”, “mostly unacceptable”, “no opinion”, “rather acceptable”, and “completely acceptable”), with higher scores indicating a more positive attitude toward suicide.

### Data analysis

In phase 1 of the study, the transcribed data were analyzed, based on the content analysis method.^26^ In both phase 2 and phase 3, exploratory factor analysis (EFA), with the Geomin rotation method, was used in Mplus version 7.4^27^ to evaluate the construct validity. As an oblique type of rotation, Geomin presents the correlations between factors (represented by asterisks), with significant factor loadings at 95% confidence interval (CI). The Kaiser-Meyer-Olkin (KMO) test and Bartlett’s test of sphericity were also used for examining the sampling adequacy and non-zero matrix of correlations, respectively.

In phase 2, the EFA consisted of two steps, where the best interpretable factor structure with an adequate fit was considered eligible (a factor loading with a lower bound of 0.32 for the first EFA and 0.40 for the second EFA).^28^ This procedure ensured that items with the best contribution to the scale were retained. For both EFAs, a high cross-loading (>0.032 and 0.40, respectively) was considered ineligible. Moreover, in phase 3, to reevaluate the construct validity of the refined scale, two-hundred randomly selected cases were entered in an EFA analysis, considering an eligible factor loading with a lower bound of 0.40. The EFA was followed by confirmatory factor analysis (CFA) with maximum likelihood (ML) estimation in Mplus version 7.4^27^ for the remaining 287 cases, as well as the total sample.

Moreover, the model fit was examined, based on the following tests: a non-significant chi-square; root mean square error of approximation (RMSEA) at 90% CI (RMSEA <0.08 as adequate and RMSEA <0.05 as excellent); *P* of close fit (PCLOSE <0.05) as a supplementary index to RMSEA; testing the null hypothesis (whether RMSEA is <0.05); comparative fit index (CFI>0.90 as adequate and CFI >0.95 as excellent); Tucker-Lewis index (TLI >0.90 as adequate and >0.95 as excellent); and standardized root mean square residual (SRMR <0.08 as adequate and <0.05 as excellent).^27,29^

Also, Pearson’s correlation coefficient test was used for correlational analysis and evaluation of convergent/divergent validity of the constructs. The assumption of normality was evaluated based on the standard skewness of <2.^30^ Also, the missing data were managed via full information ML in EFA and CFA, in addition to the expectation-maximization approach in correlational analyses. *P* value less than 0.05 was considered significant.

In terms of sample size, a minimum of 200 samples, with a minimum item-to-sample ratio of 1:5, was suggested to be adequate for the primary EFA.^31^ The results of phase 2 showed that an item-to-sample ratio of 1:6 was sufficient. Also, according to the literature,^32^ an appropriate sample size for CFA depends on the variables-to-factors ratio and the level of communalities. Overall, a variables-to-factors ratio of 5:1 at three levels of communality (low, medium, and high) requires 130 to 200 samples^32^; this criterion was met in phase 3. Finally, for correlational analysis, to reach the minimum effect size of 0.20 at 80% power and significance level of 0.05, the minimum total sample size was calculated to be 194, according to the following formula: N= [(Z_α_+Z_β_)/C]^2^ + 3, where Z_α_=1.96, Z_β_=0.84, and C=0.5*ln[(1+r)/(1-r)]=0.203.^33^ Therefore, the data obtained in the two quantitative phases were satisfactory regarding the validity of the instrument.

## Results

### Phase 1: University-specific subscale

A total of 33 codes were obtained from the content analysis, most of which were individual, relational, or contextual. The codes with at least two frequencies were considered more eligible to form meaningful categories. Afterwards, seven distinct categories were obtained based on the emerged codes, including physical health, self-development opportunities, access to academic development resources, bonding to the university, interpersonal bonds, warm and supportive atmosphere, and personal efforts and capabilities. These seven categories indicate the themes of personal, relational, and contextual factors, which reflect the framework of social-ecological resilience.^3,34^ The original wording of CYRM and ARM led to the wording of the new items. The initial draft of the questions was evaluated by and discussed with a university professor, who was the former Vice-President of Student Affairs. Two noteworthy considerations were resulted from this discussion: 1) the items were rephrased to address both the university and department as some students may feel more comfortable to engage in university activities outside their primary campus, while others may prefer to stick to their department; and 2) some prompts were added to each item to guide the respondents as to what the item may target, which was also suggested by CYRM and ARM manuals.^6,8^

### Phase 2: Pilot validation study

*Factor analysis of the original scale*

The KMO value of 0.84 indicated the adequacy of the sampling, and the result from Bartlett’s test of sphericity was significant (Approx. chi-square [741] = 3366.13, *P* < 0.001). EFA #1 was able to fit the data very well to the original scale (chi-square [270] = 453.675, *P* < 0.001; RMSEA = 0.053 [0.044, 0.061], PCLOSE = 0.273, SRMR= 0.036), indicating five items, including SER5, SER8, SER12, SER14, and SER22, with ineligible factor loadings (<0.32). Removing these five items, EFA #2, which included 25 retained items, also adequately fitted the data (Chi-square [165] = 298.373, *P* < 0.001; RMSEA = 0.061 [0.050, 0.072], PCLOSE = 0.053, SRMR= 0.036), indicating five items of SER4, SER9, SER13, SER16, and SER20 with low factor loadings (<0.40), and item SER28 with high cross-loading. Table 1 presents the results of EFA #1 and 2, showing 20 eligible items with fair to strong factor loadings on five factors of *interpersonal bonds, family support, cultural attunement, peer support, and growth opportunities.*

**Table 1:** Factor analysis of social-ecological resilience measure (main items) in phase-2 (N = 242)

**Items**	**Content**	**EFA #1**						**EFA #2**				
		**Factor1**	**Factor2**	**Factor3**	**Factor4**	**Factor5**	**Factor6**	**Interpersonal bonds**	**Family support**	**Cultural Attunement**	**Peer support**	**Growth opportunities**
SER1	People who I respect	0.597*	0.367*	-0.142	-0.010	-0.014	0.066	0.620*	0.367*	-0.045	-0.029	0.066
SER2	Co-operating with people around	0.409*	-0.040	-0.020	0.257*	0.038	0.098	0.435*	0.012	0.057	0.298*	-0.006
SER3	Importance of skill development	0.399*	0.055	-0.043	-0.001	0.411*	-0.065	0.335*	-0.016	-0.001	0.042	0.443*
SER4	Importance of education	0.341*	0.022	0.094	-0.010	0.283*	-0.118	0.314*	*-0.060*	*0.072*	*0.084*	0.258*
SER5	How to behave in social situations	0.195*	*0.054*	*0.093*	*0.060*	0.310*	*-0.040*					
SER6	Family support and protection	-0.019	0.717*	-0.155*	-0.002	-0.008	0.001	-0.003	0.713*	-0.136*	0.019	-0.032
SER7	Family know a lot about me	-0.018	0.493*	0.090	0.074	0.083	0.134	-0.052	0.498*	0.186*	0.109	0.081
SER8	Self-preparing meal	*0.154*	*0.143*	*0.017*	*0.101*	0.214*	*-0.037*					
SER9	Finishing the works	0.459*	-0.057	0.084	-0.041	0.168	0.039	0.256*	*-0.048*	*0.143*	*0.100*	*0.139*
SER10	Spiritual beliefs	0.108	0.015	0.505*	-0.058	-0.062	0.303	0.199*	0.117	0.626*	-0.040	-0.093
SER11	Ethnic background	0.100	0.027	0.201*	0.026	-0.033	0.483*	0.142	0.090	0.463*	-0.054	0.005
SER12	People feel happy with me	0.202*	-0.025	0.025	0.150	0.234*	0.106					
SER13	Talking about my feelings with family	0.052	0.330*	0.127	0.147	-0.006	0.148	*0.043*	0.292*	0.251*	0.170*	*-0.007*
SER14	Solving Problem without self-harm	0.274*	0.202*	*0.155*	*0.138*	*0.024*	*-0.158*					
SER15	Support from fiends	0.084	-0.055	-0.016	0.771*	0.003	0.005	0.117	0.008	-0.051	0.709*	0.028
SER16	Know where to look for help	0.036	0.052	0.186*	0.355*	0.119	-0.056	*-0.031*	*0.034*	*0.122*	0.369*	*0.095*
SER17	Sense of belonging to the community	-0.053	-0.015	0.738*	0.161	0.046	-0.005	-0.042	-0.075	0.657*	0.240*	0.057
SER18	Family back me through hardships	0.025	0.911*	-0.005	0.036	-0.153*	-0.055	-0.043	0.842*	-0.002	0.111	-0.071
SER19	Friends back me through hardships	-0.032	0.075	-0.002	0.825*	-0.100	0.002	-0.002	0.055	-0.033	0.867*	-0.063
SER20	Fair treatment	-0.213*	0.184*	0.370*	0.221*	0.116	0.034	-0.184*	*0.037*	0.352*	0.239*	*0.114*
SER21	Opportunities to show my independence	0.206*	-0.041	0.104	0.036	0.444*	0.087	0.129	-0.058	0.143*	0.084	0.479*
SER22	Knowing my strength	0.271*	*0.021*	0.212*	*0.051*	0.308*	*-0.071*					
SER23	Participating in religious activities	0.110	-0.030	0.652*	-0.158	-0.080	0.125	0.160	-0.009	0.640*	-0.063	-0.117
SER24	Importance of serving the community	0.042	0.062	0.639*	-0.068	-0.013	0.089	-0.001	-0.005	0.504*	-0.058	0.004
SER25	Sense of security with family	-0.019	0.693*	0.084	-0.125	0.071	0.040	0.070	0.653*	0.087	-0.006	0.124
SER26	Opportunities for development	0.023	0.082	-0.048	-0.050	0.815*	0.015	0.043	0.071	-0.061	-0.144*	0.896*
SER27	Opportunities for doing	-0.059	-0.036	-0.015	0.005	0.915*	0.036	-0.104	0.066	0.025	0.016	0.787*
SER28	Enjoy family’s traditions	0.012	0.311*	-0.007	-0.037	0.125	0.642*	*0.010*	0.437*	0.349*	*-0.091*	*0.153*
SER29	Enjoying community’s traditions	-0.044	0.040	0.361*	0.039	-0.013	0.570*	0.005	0.074	0.712*	0.009	-0.091
SER30	Citizenship	-0.083	-0.020	0.583*	0.034	0.083	0.301*	-0.105	-0.049	0.691*	0.024	0.135

*Note* . Exploratory factor analysis with Geomin rotation using Mplus (Muthen and Muthen, 1998, 2012). Asterisks indicate *P* < 0.05. Italics indicate ineligible items.

*Factor analysis of USS*

A separateEFA was performed on the university-specific subscale (USS) to obtain one to three factors. The results showed adequate fit for the two-factor solution (chi-square [19] = 40.118, *P* = 0.003 <0.01; RMSEA = 0.068 [0.037, 0.097], PCLOSE = 0.146, SRMR= 0.036), while indicating items SER36 and SER37 with ineligible factor loadings. Table 2 presents the results of EFA for USS, where factor 1 is “*educational livelihood* ” and factor 2 is “an *enabling environment* .” However, all nine factors were entered into the cross-sectional study (phase 3) in order to evaluate the devised subscale.

**Table 2:** Factor analysis of university-specific subscale in phase-2 (N = 242)

**Items**	**Content**	**Factors**	
		**Educational livelihood**	**Enabler environment**
SER31	Sense of belonging to university/department		0.497*
SER32	Having support to solve the university-related issues		0.518*
SER33	Co-operation with student association/societies	0.405*	
SER34	Opportunities to develop skills at university/department	0.827*	
SER35	Sense of contribution to the ends towards science and human development	0.662*	
SER36	University contribution to physical health	0.254*	0.320*
SER37	Sense of commitment to educational responsibilities	0.291*	0.321*
SER38	University teachers support		0.711*
SER39	Ability to resolve and follow administrative issues at university/department		0.698*

*Note* . Exploratory factor analysis with Geomin rotation using Mplus (Muthen and Muthen, 1998, 2012). Asterisks indicate *P* < 0.05. Italics indicate ineligible items.

*Factor analysis of partner support subscale*

The EFA on 122 partnered participants indicated one factor with an eigenvalue of 2.173 (with a degree of freedom = 0 resulted in reporting no fit indices). The SERp1, SERp2, and SERp3 showed good to strong factor loadings of 0.868, 0.698, and 0.736, respectively.

*Reliability*

The Cronbach’s alpha coefficient for the total resilience (30 items) was 0.89, while it was 0.85 for adjusted total resilience (only including items retained in EFA #2, Table 1), and 0.83 for total USS. Except for interpersonal bonds with a Cronbach’s alpha coefficient of 0.53, the other resilience subscales showed sufficient internal consistency from 0.73 to 0.83.

*Convergent/divergent validity*

Table 3 shows the correlations between resilience variables and depression, anxiety, and stress. The total resilience and the adjusted total resilience had extremely high correlation (r = 0.97, *P* < 0.001). Moreover, there were some positive and significant correlations between each pair of the main subscales (*P* < 0.05 to *P* < 0.001), except for partner support with family support (*P* = 0.193) and educational livelihood (*P* = 0.779). Moreover, all main resilience variables showed expected negative and significant correlation with depression (*P* < 0.05 to *P* < 0.001), except for partner support (*P* = 0.064), and to a lesser degree with stress (*P* < 0.05 to *P* < 0.001), except for university-related variables (*P* = 0.144 to 0.250) and partner support (*P* = 0.183). Only total resilience (*P* < 0.01), adjusted total resilience (*P* < 0.05), family support (*P* < 0.01), and growth opportunities (*P* < 0.05) indicated positively significant relationships with anxiety. These results indicated the convergent/divergent validity of all resilience variables and, to a lesser extent, partner support (n = 122).

**Table 3:** Pearson’s correlation matrix between study variables in phase-2 (N = 242)

	**Variables**	**Alpha**	**Mean (SD)**	**Skewness**	**Kurtosis**	**1**	**2**	**3**	**4**	**5**	**6**	**7**	**8**	**9**	**10**	**11**	**12**	**13**	**14**
1	Total resilience	0.89	3.60 (0.48)	-0.20	0.05	1													
2	Adjusted total resilience	0.85	3.62 (0.53)	-0.23	-0.07	0.97^***^	1												
3	Interpersonal bonds	0.52	4.07 (0.70)	-1.16	2.32	0.50^***^	0.49^***^	1											
4	Family support	0.78	3.83 (0.76)	-0.65	0.17	0.67^***^	0.73^***^	0.33^***^	1										
5	Culture attunement	0.83	3.23 (0.84)	-0.40	-0.16	0.75^***^	0.81^***^	0.19^***^	0.38^***^	1									
6	Peer support	0.78	3.35 (0.84)	-0.34	-0.16	0.45^***^	0.43^***^	0.25^***^	0.25^***^	0.17^**^	1								
7	Growth opportunities	0.79	3.94 (0.70)	-0.56	0.14	0.64^***^	0.60^***^	0.34^***^	0.31^***^	0.27^***^	0.19^**^	1							
8	Total university-specific	0.83	3.06 (0.75)	-0.10	-0.27	0.58^***^	0.55^***^	0.22^**^	0.28^***^	0.43^***^	0.30^***^	0.52^***^	1						
9	Educational livelihood	0.73	2.75 (0.95)	0.07	-0.54	0.43^***^	0.43^***^	0.16^*^	0.16^*^	0.37^***^	0.24^***^	0.40^***^	0.87^***^	1					
10	Enabler environment	0.73	3.25 (0.87)	-0.12	-0.22	0.55^***^	0.51^***^	0.22^***^	0.27^***^	0.37^***^	0.28^***^	0.50^***^	0.89^***^	0.56^***^	1				
11	Partner support (n=122)	0.81	3.67 (0.95)	-0.59	0.22	0.36^***^	0.31^**^	0.35^***^	0.12_.193_	0.20^*^	0.23^*^	0.31^**^	0.03_.779_	0.28^**^	0.19^*^	1			
12	Depression ( n = 239)	-	13.59 (10.97)	0.93	0.47	-0.42^***^	-0.41^***^	-0.21^**^	-0.38^***^	-0.23^***^	-0.24^***^	-0.29^***^	-0.14^*^	-0.21^**^	-0.21^**^	-0.17_.064_	1		
13	Anxiety (n = 239)	-	10.57 (9.33)	1.48	2.98	-0.17^**^	-0.16^*^	-0.09_.151_	-0.20^**^	-0.05_.449_	-0.12_.076_	-0.14^*^	-0.02_.351_	-0.09_.735_	-0.06_.166_	-0.14_.135_	0.74^***^	1	
14	Stress (n = 240)	-	17.33 (10.24)	0.86	0.87	-0.28^***^	-0.26^***^	-0.13^*^	-0.25^***^	-0.15^*^	-0.14^*^	-0.17^**^	-0.08_.250_	-0.10_.144_	-0.09_.156_	-0.12_.183_	0.81^***^	0.80^***^	1

*Note.* * *P* < 0.05, ** *P* < 0.01, *** *P* < 0.001. The lower scripts denote the actual *P* value for non-significant coefficients (*P* > 0.05) SD: Standard deviation. Alpha denotes Cronbach’s alpha coefficient. Total resilience is the sum of the main subscales and university-specific subscale, excluding partner support. Main resilience is the sum of the main subscales, including family support, peer support, and growth opportunities.

### Phase-3: Cross-validation study

*EFA*

The EFA for the cross-validation study was conducted on a combination of 21 main items (including a devised item for peer support) and nine items from the USS. From the data obtained from 487 subjects in phase-3, data of 200 subjects were randomly selected for the EFA, and the remaining 287 data were reserved for CFA. The KMO value of 0.81 indicated the sampling adequacy, and the result of Bartlett’s Test of Sphericity was significant (Approx. chi-squared [465] = 2773.50, *P* < 0.001). The EFA was defined to derive 5-7 factors. The five-factor solution showed the best interpretability as well as adequate fit (Chi-square [295] = 621.061, *P* < 0.001; RMSEA = 0.074 [0.066, 0.082], PCLOSE <0.001, SRMR= 0.042). Table 3 presents the results of the EFA, confirming the results of the previous EFA in phase 2 for the original scale. However, items SER11 and SER1 did not influence the function of robust estimators on their alleged factors. Also, UNI1 and UNI7 did not show adequate factor loadings on USS. Table 4 presents the details of the EFAs.

**Table 4:** Factor analysis of main measure and university-specific subscale

**Items**	**EFA (n = 200)** ^a^					**CFA Factor Loadings (n = 287)** ^b^	**CFA Factor Loadings (N = 487)** ^b^
	**Family support**	**Peer support**	**Cultural attunement**	**Growth opportunities**	**University-specific**		
**Main measure**							
SER6	0.764*					0.811*	0.798*
SER7	0.724*					0.633*	0.661*
SER13	0.703*					0.574*	0.613*
SER18	0.816*					0.756*	0.787*
SER25	0.740*					0.785*	0.785*
SER15		0.805*				0.862*	0.843*
SER19		0.883*				0.856*	0.880*
SERpeer		0.548*				0.571*	0.572*
SER10			0.581*			0.547*	0.577*
SER11			0.308*			0.418*	0.400*
SER17			0.697*			0.710*	0.737*
SER23			0.623*			0.529*	0.590*
SER24			0.710*			0.603*	0.681*
SER29			0.724*			0.634*	0.636*
SER30			0.794*			0.718*	0.728*
SER1				0.254*		0.530*	0.531*
SER2				0.464*		0.358*	0.446*
SER3				0.548*		0.465*	0.562*
SER21				0.480*		0.508*	0.468*
SER26				0.900*		0.497*	0.491*
SER27				0.770*		0.546*	0.467*
**University-specific subscale**							
UNI1					0.343*	**0.583**	**0.561**
UNI2					0.563*	**0.614**	**0.590**
UNI3					0.406*	**0.566**	**0.543**
UNI4					0.650*	**0.711**	**0.717**
UNI5					0.767*	**0.671**	**0.685**
UNI6					0.530*	**0.462**	**0.463**
UNI7					0.247*	**0.481**	**0.478**
UNI8					0.405*	**0.508**	**0.534**
UNI9					0.409*	**0.536**	**0.545**
Eigenvalues	7.606	3.378	2.521	1.975	1.583		

*Note* . Asterisks indicate *P* <0.05. EFA: Exploratory factor analysis. CFA: Confirmatory factor analysis. SER denotes social-ecological resilience items. SERpeer denotes the new item added to the peer support subscale. UNI denotes items of the university-specific subscale. The items are numbered as Table 1. ^a^EFA on the full measure, including main subscales and university-specific subscale. ^b^CFA separately on main subscales and university-specific subscale.

*CFA*

The suggestions of the previous EFA were reevaluated by two CFAs on the remaining phase-3 subsample (n = 278) and the total sample (n = 487). Table 4 presents the factor loadings derived from the two CFAs. Furthermore, the CFA on the full measure (including USS) showed an unsatisfactory fitness. Therefore, two CFAs were followed up on the refined main measure, including family, peers, culture, and growth, and separately on the USS. For 287 remained samples, the model, including five eligible error covariances, indicated marginal fit (chi-square [178] = 426.663, *P* < 0.001; RMSEA = 0.070 [0.061, 0.078], PCLOSE = 0.001, CFI = 0.891, TLI = 0.871, and SRMR = 0.069). All indicators significantly contributed to the factors with fair to excellent loadings. In the total sample, the model fit improved via two additional error covariances (chi-square [176] = 515.413, *P* <0.001; RMSEA = 0.063 [0.057, 0.069], PCLOSE < 0.001, CFI= 0.918, TLI = 0.902, and SRMR = 0.058), indicating fair to excellent factor loadings (See Supplementary file 1, Figure S1). Moreover, the CFA on USS, including three eligible error covariances, indicated adequate model fit (Chi-square [24] = 68.656, *P* <0.001; RMSEA = 0.062 [0.045, 0.072], PCLOSE = 0.119, CFI = 0.957, TLI = 0.936, and SRMR = 0.035). Strategically, as SERT suggested, two variables of personal growth (items SER1, 2, 3) and growth opportunities (SER 26, 27, 28) were forged to evaluate the specific nature of individual-oriented growth tendencies and context-oriented growth opportunities (See Supplementary file 1, Figure S2).

*Partner support subscale*

The cross-validation CFA among 131 partnered participants confirmed the one-factor model (with a degree of freedom = 0, which resulted in reporting no fit indices) with factor loadings of 0.841, 0.608, and 0.779 (α = 0.78).

*Reliability*

Table 5 reports the internal consistency of the resilience variables. The full measure (including USS) and the refined main measure showed Cronbach’s alpha coefficients of 0.89 and 0.86, respectively, while the Cronbach’s alpha coefficients for the subscales ranged from 0.66 to 0.86. The results indicated sound internal consistency for the variables.

*Convergent/divergent validity*

Table 5 presents the correlations between resilience variables, depression, anxiety, FoL, and SA. There were expected positive and significant correlations between each pair of resilience variables (*P* < 0.001), except for partner support with peer support (*P* = 0.214) and growth opportunities (*P* = 0.055). Also, except for personal growth with anxiety (*P* = 0.206) as well as partner support with depression (*P* = 0.084), all resilience variables showed expected negative and significant relationships with depression (*P* < 0.001) and anxiety (*P* < 0.01 to *P* < 0.001). Also, total resilience (including USS) and main resilience (excluding USS) showed expected negative and significant correlations with PAP, FoL, and SA (*P* < 0.001). All subscales had positive and significant correlation with PAP (*P* < 0.05 to *P* < 0.001) and negative correlation with FoL (*P* < 0.01 to *P* < 0.001). However, peer support (*P* = 0.244), personal growth (*P* = 0.092), and partner support (*P* = 0.444) showed no significant correlation with SA. The results supported the convergent and divergent validity of the resilience variables. The Supplementary file 2 presents the content and scoring procedure of the refined Student-SERM.

**Table 5:** Pearson’s correlation matrix between study variables in phase-3 (N = 487)

**Variables**		**Alpha**	**Mean (SD)**	**Skewness**	**Kurtosis**	**1**	**2**	**3**	**4**	**5**	**6**	**7**	**8**	**9**	**10**	**11**	**12**	**13**	**14**	**15**
1	Total resilience	0.89	3.37 (0.54)	0.00	-0.14	1														
2	Main resilience	0.86	3.50 (0.57)	-0.01	-0.15	0.97^***^	1													
3	Family support	0.86	3.74 (0.85)	-0.56	-0.12	0.68^***^	0.73^***^	1												
4	Peer support	0.81	3.20 (0.92)	-0.16	-0.44	0.63^***^	0.67^***^	0.28^***^	1											
5	Cultural attunement	0.81	3.10 (0.89)	0.11	-0.52	0.69^***^	0.70^***^	0.38^***^	0.19^***^	1										
6	Personal growth	0.66	4.39 (0.61)	-1.25	2.46	0.55^***^	0.54^***^	0.34^***^	0.20^***^	0.30^***^	1									
7	Growth opportunities	0.79	3.52 (0.87)	-0.31	-0.17	0.60^***^	0.56^***^	0.24^***^	0.32^***^	0.23^***^	0.42^***^	1								
8	Growth total	0.76	3.95 (0.63)	-0.67	1.18	0.69^***^	0.65^***^	0.33^***^	0.32^***^	0.31^***^	0.76^***^	0.90^***^	1							
9	University-specific	0.81	2.84 (0.75)	0.16	-0.07	0.70^***^	0.50^***^	0.25^***^	0.26^***^	0.40^***^	0.36^***^	0.50^***^	0.52^***^	1						
10	Partner support (n=131)	0.78	3.76 (1.0)	-0.77	-0.07	0.31^***^	0.29^**^	0.27^**^	0.11_.214_	0.19^*^	0.30^**^	0.17_.055_	0.27^**^	0.22^*^	1					
11	Depression	-	5.95 (4.15)	0.77	-0.10	-0.52^**^	-0.51^**^	-0.39^**^	-0.30^**^	-0.35^**^	-0.24^**^	-0.33^**^	-0.35^**^	-0.36^**^	-0.15_.084_	1				
12	Anxiety	-	9.22 (4.32)	0.14	-0.49	-0.31^**^	-0.31^**^	-0.28^**^	-0.15^**^	-0.24^**^	-0.06_.206_	-0.19^**^	-0.16^**^	-0.21^**^	-0.21^*^	0.60^**^	1			
13	PAP	-	3.43 (0.88)	-0.27	0.03	0.27^***^	0.24^***^	0.18^***^	0.12_.011_^*^	0.15_.001_^**^	0.20^***^	0.21^***^	0.24^***^	0.26^***^	0.17^*^	-0.32^**^	-0.17^**^	1		
14	FoL	-	2.83 (1.14)	0.31	-0.79	-0.38^***^	-0.39^***^	-0.39^***^	-0.25^***^	-0.23^***^	-0.13_.003_^**^	-0.17^***^	-0.18^***^	-0.21^***^	-0.44^***^	0.45^***^	0.43^***^	-0.20^***^	1	
15	SA	-	2.24 (1.30)	0.48	-1.28	-0.30^***^	-0.31^***^	-0.28^***^	-0.05_.244_	-0.38^***^	-0.08_.092_	-0.11^*^	-0.11^*^	-0.17^***^	-.07_.444_	0.30^***^	.31^***^	-0.01_.867_	0.30^***^	1

Note. *P <0.05, ** *P* <0.01, *** *P* <0.001. The lower scripts denote the actual *P* values for *P* >0.05. SD: Standard deviation. PAP: perceived academic performance. FoL: Feeling of loneliness. SA: Suicide acceptability. Two messing data for FoL and one missing datum for SA.

## Discussion

This study primarily supported the composition of SERT reflected the Student-SERM (based on SERT), including family support, peer support, cultural attunement, and growth dimensions, which consistently constituted the ecological context for the youth and contributed to their resilience.^3,4^ Different dimensions of resilience, especially familial, educational, and cultural aspects, steadily emerge as the main areas of concern for the university students’ well-being.^35^ Although CYRM primarily considers the importance of individual skills, including personal skills, peer support, and social skills,^6^ the current study only retained education-related behaviors in the Student-SERM, including “having people to respect”, “importance of skill development”, and “cooperation with others”. However, behavioral skills, that is, “showing appropriate behaviors” and “knowing one’s strengths”, could not eligibly contribute to any factors in phase 2. Despite the possible effect of sample size on the results, phase 2 indicated the interrelatedness of all sets of items, except for personal skills.

Also, an extremely high correlation was found between the total resilience (30 items) and the adjusted score (20 items), which supported the reduction of the scale. However, in CYRM, the relevance of personal skills in young populations was originally confirmed.^5^ Therefore, one might arguably suggest that socio-ecological resilience must be independently investigated during the individual’s transition into adulthood to identify the skills and behaviors in action. On the other hand, this study supported the importance of education-related, personal capacity in shaping the students’ resilience.

The centrality of family in the students’ mental health has been consistently described in the literature.^14,36^ From an ecological perspective, the parents’ capabilities (e.g., educational level) and family economic status seem to influence the students’ well-being and self-efficacy.^37-40^ Therefore, contribution of family to the students’ resilience may not solely depend on interpersonal bonds, but may be rather attributed to provisions and opportunities that are either offered by the family or the broader socioeconomic context. Also, some other studies have indicated the role of friends, besides family, in the university students’ well-being.^41^ Some studies, by adopting an individualistic approach to resilience, have shown the interactive effects of peer support and personal strength in shaping the young people’s competencies.^42^ It should be noted that the Student-SERM entails a new item, addressing the students’ communication with their peers about their problems. Although this particular item had a lesser contribution to the peer support factor, it showed the extent to which the student could approach his/her peers in face of hardship.

Moreover, the present results indicated the growth subscale as a single factor, while some functional differences were observed between personal growth tendencies and contextual opportunities. The contextual opportunities had significant associations with anxiety and SA, while personal growth did not show any correlations. Therefore, more opportunities for self-development and independence are associated with less anxiety and suicidal attempts in students. Also, this finding represents an impaired relationship between the student and the immediate educational setting, which must satisfy his/her developmental needs for a better future. Overall, the present results suggested that the students’ well-being is somewhat influenced by their satisfaction with the availability and accessibility of contextual resources.

The current study also developed and proposed USS as a site-specific subscale,^6,8^ according to the students’ viewpoints. The USS encompasses the individual’s activity level and contextual capabilities, aligned with SERT. Besides, USS not only addresses personal skills and social engagement as behaviors specific to academic settings, but also ties the resilience construct to a sense of contribution to human development at large. In other words, it characterizes academic achievements as a “spiritual enterprise”. Also, it is worth mentioning that “livelihood”, which refers to the student’s level of activity and engagement in the university setting, stems from a network of relationships with peers and faculty members.^43^ Moreover, the influence of university instructors as enabling mentors contributes to the students’ capabilities.^44^ Likewise, contextual factors, including peer support, family support, institutional facilities, and community values, besides extracurricular activities, may affect the student assessment.^45^ Universities fundamentally constitute the quality of student development in terms of various personal, social, cultural, and educational aspects.^46^ Therefore, they may partly shape the students’ perceptions, which influence their academic performance (i.e., a deep or surface approach to learning)^47^ and educational outcomes. Since the educational outcomes are believed to be both individual and environmental,^48^ the Student-SERM critically addresses the role of immediate educational context in manifesting adequate resilience in student populations.

The current study also reported culture attunement as a single factor. Generally, cultural resources can majorly influence the student’s life in different ways.^35,49,50^ Cultural attunement addresses the individual’s contentedness and satisfaction with the customs, ceremonies, and religious, social, and ethnic practices.^6,51^ However, some may argue that the developed items only capture the mainstream cultures. In other words, the construct may indicate how much the individual is satisfied with the dominant community’s cultural patterns, but may not show the extent to which his/her specific cultural demands are met in a given social context. For example, this study indicated that cultural attunement had the strongest negative correlation with SA, compared to other resilience factors. Therefore, the operational definition of this construct allows seemingly contradictory interpretations of whether it is necessary to encourage the individual to accept his/her community’s cultural practices or focus on improving the contextual context, as implied by SERT.

Finally, the present study supported the configuration of SERT in a young Iranian population in a university setting and offered a holistic approach toward the resilience construct. However, the resilience construct in Iranian youth is mostly limited to an individual level.^19-21^ Although some recent studies on adolescent students have applied CYRM,^52^ and some have incorporated family, peer, and community/school dimensions in resilience measures,^53^ it seems that more emphasis should be placed on the ecological context in resilience research.^34^ This critique may be more illustrated in a recent study,^54^ which showed that a newly designed tool for adolescents after adversity failed to capture the availability and accessibility of empowering resources in the ecological context.^2,3^ Therefore, the Student-SERM may promote ecological insights into the resilience process among student populations.

### Limitations

This study was subjected to some limitations. Firstly, the response bias and social desirability bias, inherent to the self-report method of assessment, might jeopardize the accuracy of responses. Secondly, since the study sample was only selected from one university in Tehran, Iran, our findings cannot be generalized to the entire Iranian student population; therefore, there is a need to support the present results in a multi-center cross-validation study. Thirdly, the causal relationships could not be inferred considering the study design. Fourthly, although some studies have suggested the validity of single-item measures for assessing mental health in population-based reserach,^55^ convergent and divergent validity might not indicate a rigorous construct to establish the criterion-related validity of the scale. Therefore, the current results should be considered as primary findings regarding the construct validity of the Student-SERM, and further information is required for confirmation.^56^ Finally, researchers should apply longitudinal or interventional research designs to verify SERT in the future and to evaluate the functionality of the Student-SERM in university settings among students facing adverse events.

## Conclusion

The current study, which was based on SERT,^2,34^ indicated that the Student-SERM is a valid instrument for assessing resilience factors. However, further research is needed to confirm the stable factor structure of this instrument in different student populations and to study its application in high-risk students. Our newly developed tool encompassed the socio-ecological context of undergraduate students, including family support, peer support, cultural attunement, growth (i.e., educational attainment and contextual opportunities for growth), and university-specific factors. Based on the present results, the Student-SERM can be considered a valid resilience tool, which surpasses the individual level of resilience. It also embraces the students’ broad ecological context, allowing them to manifest resilience during their study period and life in general.

## Ethical approval

This research was approved by the deputy of Iran National Committee for Ethics in Biomedical Research, University of Tehran, Iran (ID: IR.UT.PSYEDU.REC.1397.002). Informed consent was obtained from all participants.

## Competing interests

The authors declare that they have no conflict of interest.

## Funding

This study was derived from Suicidality-RP Factors Project, which was funded the Research Committee of Promoting Students’ Mental Health, University of Tehran, Tehran, Iran (#6009818). The University of Tehran’s Counseling Center supported the study non-financially.

## Authors’ contributions

MAT and HZ contributed to the study conception and design. Material preparation and data collection for the phase-2 were performed by MN, ESH, and MAT. Material preparation and data collection for the phase-3 were performed by MN, RS, TJ, and MAT. Analyses were performed by MAT under the supervision of HZ. The first draft of the manuscript was written by MAT and RS. All authors commented on previous versions of the manuscript. All authors read and approved the final manuscript.

## Acknowledgments

The authors would like to express their gratitude to Prof. Michael Ungar for granting the permission for developing the Student-SERM as an adaptation of CYRM-28 and RRC-ARM. We are also thankful to Mr. Mostafa Akbari for his kind collaboration in the study. We would like to express our deepest gratitude to the participants for their time and engagement.

## Supplementary Materials


Supplementary file 1 contains Figures S1 and S2.

Supplementary file 2 contains the content and the scoring procedure of Student-SERM.
