# Supplementary material for: Social-Ecological measure of resilience: an adapted measure for Persian-speaking university students
Source: Health Promot Perspect. 2020 Jul 12;10(3):207–19. doi: 10.34172/hpp.2020.34 (PMC7420169; doi:10.34172/hpp.2020.34)
Supplement: Supplementary file 1 — contains Figures S1 and S2. [file hpp-10-207-s001.pdf]

Supplementary file 1

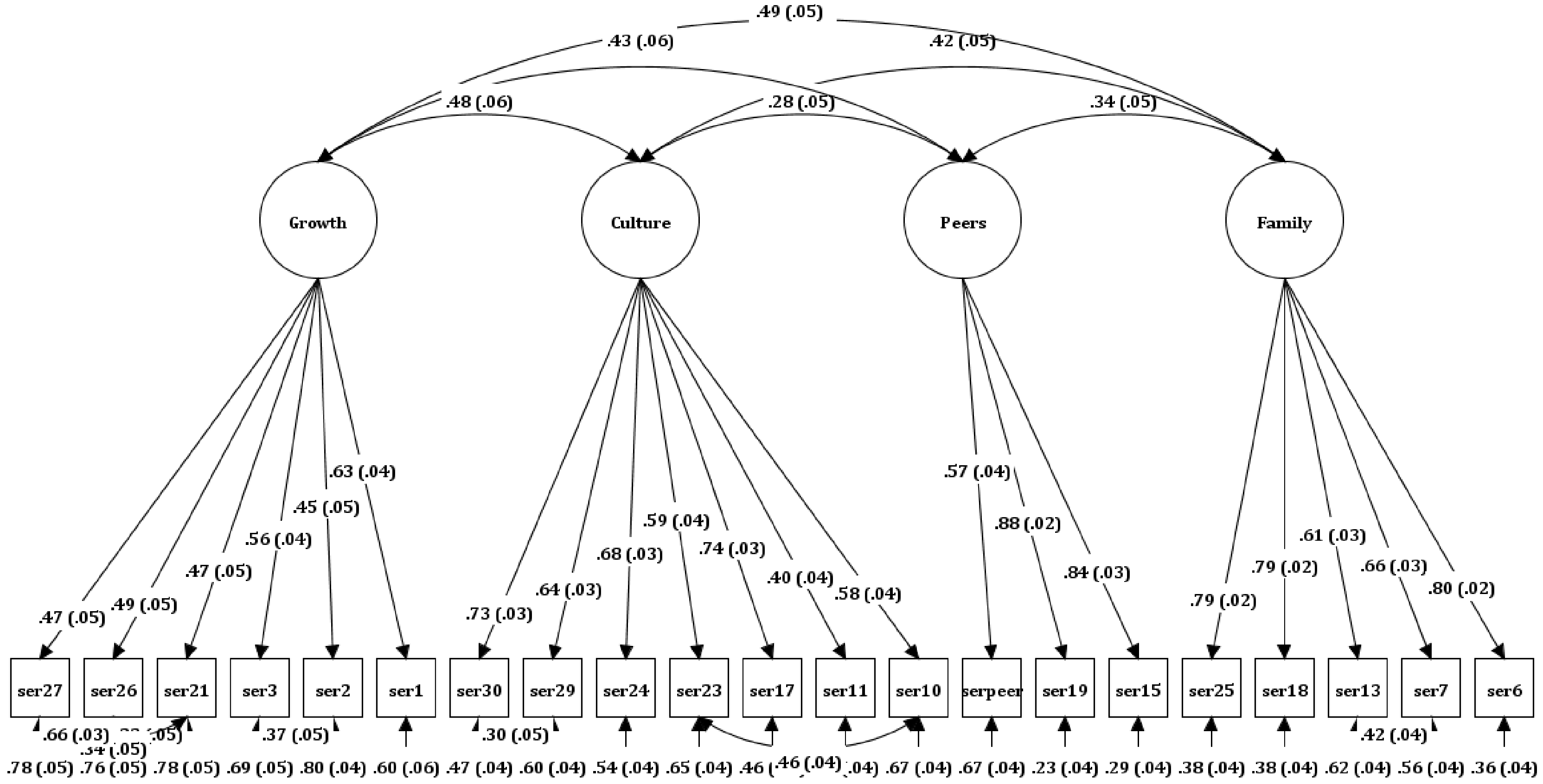

Figure S1. Confirmatory factor analysis with ML estimation on the main subscales of the Student-SERM in the phase-3 (N = 487, Chi-squared [176] = 515.413, *P* <0.001; RMSEA = 0.063, 90% Confidence interval: [0.057, 0.069], PCLOSE <0.001, CFI= 0.918, TLI= 0.902, and SRMR= 0.058).

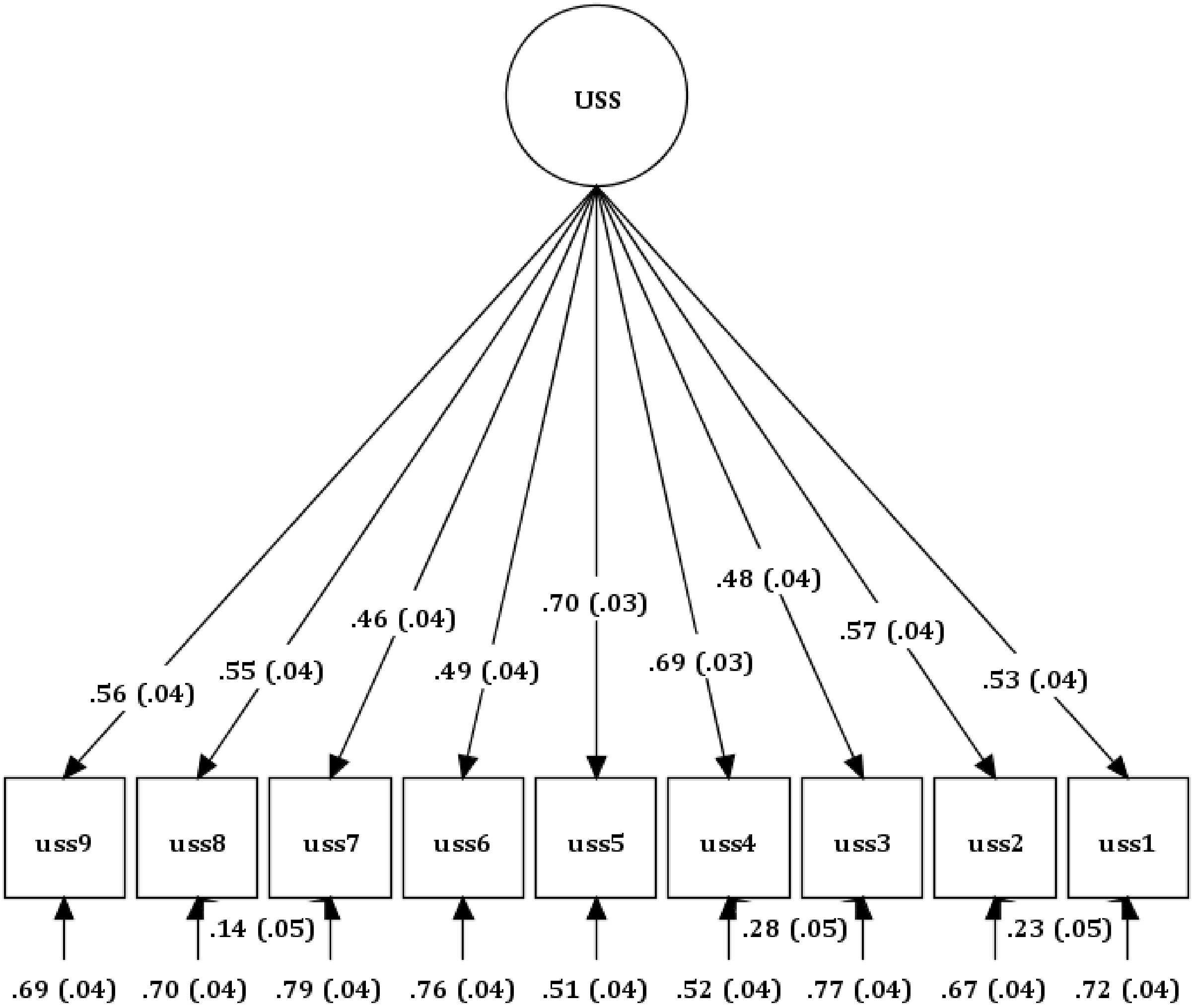

Figure S2. Confirmatory factor analysis with ML estimation on the university-specific subscale (USS) of the Student-SERM in the phase-3. (N = 487, Chi-squared [24] = 68.656, P <0.001; RMSEA = 0.062, 90% Confidence interval: [0.045, 0.072], PCLOSE = 0.119, CFI= 0.957, TLI= 0.936, and SRMR= 0.035).
