# Supplementary material for: Social-Ecological measure of resilience: an adapted measure for Persian-speaking university students
Source: Health Promot Perspect. 2020 Jul 12;10(3):207–19. doi: 10.34172/hpp.2020.34 (PMC7420169; doi:10.34172/hpp.2020.34)
Supplement: Supplementary file 2 — contains the content and the scoring procedure of Student-SERM. [file hpp-10-207-s002.pdf]

Supplementary File 2

The Student Social-Ecological Resilience Measure (Student-SERM)		Type
**Family support**		***Scale***
SER6	Family support and protection	***Ordinal***
SER7	Family know a lot about me	***Ordinal***
SER13	Talking about my feelings with family	***Ordinal***
SER18	Family back me through hardships	***Ordinal***
SER25	Sense of security with family	***Ordinal***
**Peer support**		***Scale***
SER15	Support from friends	***Ordinal***
SER19	Friends back me through hardships	***Ordinal***
SERpeer	Talking about my feelings with friends	***Ordinal***
**Cultural attunement**		***Scale***
SER10	Spiritual beliefs	***Ordinal***
SER11	Ethnic background	***Ordinal***
SER17	Sense of belonging to the community	***Ordinal***
SER23	Participating in religious activities	***Ordinal***
SER24	Importance of serving the community	***Ordinal***
SER29	Enjoying my community's traditions	***Ordinal***
SER30	Being proud of citizenship	***Ordinal***
**Growth opportunities**		***Scale***
SER1	People whom I respect	***Ordinal***
SER2	Co-operating with people around	***Ordinal***
SER3	Importance of skill development	***Ordinal***
SER21	Opportunities to show my independence	***Ordinal***
SER26	Opportunities for development	***Ordinal***
SER27	Opportunities for doing	***Ordinal***
**University-specific subscale (USS)**		***Scale***
UNI1	Sense of belonging to university/department	***Ordinal***
UNI2	Having support to solve the university-related issues	***Ordinal***
UNI3	Co-operation with student association/societies	***Ordinal***
UNI4	Opportunities to develop skills at university/department	***Ordinal***
UNI5	Sense of contribution to the ends towards science and human development	***Ordinal***
UNI6	University contribution to physical health	***Ordinal***
UNI7	Sense of commitment to educational responsibilities	***Ordinal***
UNI8	University teachers support	***Ordinal***
UNI9	Ability to resolve and follow administrative issues at university/department	***Ordinal***
**Partnership**	**(1) Married (2)Casual partnership (3) No Partnership**	***Nominal***
**Partner support**		***Scale***
PAR1	Talking about my feelings to my spouse/partner	***Ordinal***
PAR2	Getting support from my spouse/partner during difficulties	***Ordinal***
PAR3	Enjoying my spouse/partner's culture and tradition	***Ordinal***

Note:

All the items are rated based on 1 to 5 Likert-type scaling, including never (1), low (2), somewhat (3), quite a bit (4), and a lot (5). There is no reverse Item.

The original measures may be found through the links below:

Resilience Research Centre. The Child and Youth Resilience Measure (CYRM) Youth Version. Dalhousie University, Canada; 2016. http://www.resilienceresearch.org.

Resilience Research Centre. The Resilience Research Centre Adult Resilience Measure (RRC-ARM). Dalhousie University, Canada; 2016. http://www.resilienceresearch.org.

The Persian version of the instrument is accessible via contact with the corresponding author at amini.m.ali@ut.ac.ir
